# Attitudes towards teachers supporting student mental health in rural contexts: a pilot study examining community perspectives

**DOI:** 10.3389/fpsyt.2025.1613341

**Published:** 2025-10-27

**Authors:** Brian Moore, Sarah Redshaw, Nicole Masters, Erin Mackenzie, Roberto H. Parada, Lena Danaia

**Affiliations:** 1 School of Education, University of Wollongong, Wollongong, NSW, Australia; 2 School of Education, Charles Sturt University, Bathurst, NSW, Australia; 3 School of Education, Western Sydney University, Sydney, NSW, Australia

**Keywords:** mental health, schools, students, teachers, wellbeing

## Abstract

**Introduction:**

While the prevalence and burden of mental health issues continues to rise, access to mental health support services for children and adolescents is under resourced, particularly in rural settings. Consequently, school teachers are increasingly being positioned to assume mental health support roles to address this problem, which the Australian Government Productivity Commission has referred to as a community expectation.

**Methods:**

This pilot study aimed to investigate community expectations regarding teachers supporting student mental health in rural contexts, employing a cross-sectional, mixed-methods design using a brief survey. Fifty-seven participants including parents and caregivers, community members, mental health practitioners, and teachers completed the survey.

**Results:**

The results did not consistently support the idea that teachers should be expected to do mental health work to support students in rural schools, but clearly supported the view that teachers are not adequately trained to perform mental health work with students.

**Discussion:**

Although the study provides some support for the community expectation that teachers have a role in supporting student mental health in schools, this is not a consistently held community view. Further clarification of this, with particular reference to defining the teacher’s role in mental health services in schools is needed.

## 1 Introduction

Mental health is a major societal issue with an estimated annual global cost of US$2.5 trillion (1). As a significant proportion of mental health conditions onset prior to 18 years of age (2), early intervention is critical for addressing the longer-term impacts of poor mental health. However, in the Australian context mental health and psychiatric care are “grossly” underfunded (3, para 2). Consequently, according to the Australian Government Productivity Commission [AGPC] (4) the community increasingly expects school teachers to perform frontline mental health provider roles, despite little training or resources (5). However, there appears to be little data supporting this reported expectation.

### 1.1 Defining mental health

A range of terms related to mental health are used interchangeably, such as mental disorder, mental illness, mental health problems, mental health difficulties, having poor mental health, mental ill health, mental health conditions, and experiencing psychological distress. However, “mental health is more than the absence of mental disorders” (6, para 2) and should also consider the social, emotional, and behavioural well-being of individuals (7). This paper follows the definition offered by the WHO (6) which defines mental health on a continuum, ranging from mental illness to wellbeing. Mental health refers to mental states in which an individual has the capacity to cope with the complexities of life, exercise agency, and importantly can effectively learn, work and contribute to their community. It is important to note that this continuum is complex and “is experienced differently from one person to the next, with varying degrees of difficulty and distress and potentially very different social and clinical outcomes” (6, para 2).

### 1.2 Mental disorders (or ill-health) in children and adolescents

Mental disorders frequently onset during early childhood and adolescence (2) and the WHO reported that half of all mental health disorders experienced in adulthood begin by age 14 (8). The need for mental health services for school‐age children appears to be increasing (9) with psychological disorders accounting for 45% of the global burden of disease among young people aged between 10–24 years (10). In the US, an estimated 20-25% of children experience mental health disorders each year (11). There is an increasing body of research highlighting the incidence of a range of debilitating mental health problems in children and young people across Australia (12) and globally (13).

While it is reported that one in seven young Australians aged 4–17 years are diagnosed with a mental illness (12), there is currently no regular national system for monitoring and reporting on the overall mental health and wellbeing of children aged 0–12 years (14). As a result, there is a disparity in statistics reporting mental health difficulties in children and variable percentages of school-aged students experiencing mental health issues (15). Nonetheless, it is important to note that particular categories of young people appear more likely to experience mental health issues including those identifying as female (16), experiencing disability or a chronic illness (17), reporting adverse childhood experiences (18), identifying as transgender and gender diverse (19), who have recently migrated or are refugees (20), whose parents or caregivers live with mental illness (21), who have experienced the child protection system (18), or those who have been incarcerated as part of the youth justice system (18).

The mental health of Australian youths residing in remote and rural areas is of particular concern. A recent study reported that one in four rural youth aged 15–19 years had a serious mental illness (22) and noted serious mental illness was almost twice as likely for females. Suicide rates are higher for rural youth (22) with some research reporting the rates of death by suicide for males residing in rural areas are nearly double when compared with males who reside in urban areas (23).

Despite the significant prevalence of mental illness in youth, only 30% of Australian adolescents aged 13–17 years report using mental health services for support (24), and younger children aged 4–11 years were even less likely to access a mental health service (12). Young people have reported multiple barriers to accessing mental health services including poor mental health literacy, fear of being stigmatised, a preference for self-management and informal support, expense, and the accessibility of mental health treatment (12, 16, 18, 25). These barriers are particularly difficult for youth residing in rural locations (23).

Service accessibility may be especially problematic for youth living in rural communities. Around 30% of all Australian children aged 0–14 years live in rural areas, and 3% live in remote and very remote areas (18). For many children and adolescents, the nearest services are located in major hospitals a long distance away, which requires a lengthy trip to see a service provider in person (26), although it should be noted that Telehealth and online services have rapidly grown in rural settings since the COVID-19 pandemic (27). While mental health service accessibility is an ongoing problem nationally, rural contexts are more challenging settings for providing mental health care given the shortage of relevant health services in rural Australia (28). For example, in NSW there are 24.4 psychiatrists per 100,000 people in major and inner regional cities, whereas there are.09 in outer regional, remote, and very remote areas; with similar ratios for psychologists and other mental health professionals (29). It is likely that these numbers are overestimated if we consider that they refer to generalist and not child and adolescent specialist mental health services (30).

### 1.3 The school context and the role of teachers supporting mental health

In Australia, the education system is broadly structured to provide 7 years of primary education and 6 years of secondary education, which can be delivered by public, Catholic, or privately funded schools. As schools generally provide universal education services to youth (31) they are often positioned as a setting for promoting youth mental health and wellbeing (14). While schools are primarily concerned with education, there is a strong association between students’ learning, academic progress, and mental wellbeing (32) as well as social and emotional development (33). However, although many students rely on school-based services as they do not have access to community-based mental health providers (34), mental health promotion in schools is under resourced (35) and most school-based efforts are reactive rather than proactive (33).

In Australia, the Black Dog Institute^1^ has noted that community expectations of teachers as frontline mental health providers who provide care for their students’ mental health and wellbeing has grown stronger since COVID-19 (36). However, it is important to note that the role of teachers in promoting wellbeing and preventing mental ill-health has never been clearly articulated by Australian governments (4). Further, many pre-service teacher education programs do not explicitly address mental health training (37), which may not occur at all (38). This may explain why many teachers report low confidence to carry out roles in mental health care (39). Given that experienced mental health professionals report feelings of incompetence when addressing their clients’ mental health needs (40), community expectations of teachers in the mental health space should be carefully considered (41).

Australian policy reports have recommended that schools be leveraged to support student mental health in regional and rural areas more effectively, however a very limited body of research has examined this space (42). While students at rural schools report valuing mental health programs provided at schools, it was noted that school-based services such as school counselling were “extremely under resourced and not meeting demand” (43, p.5).

### 1.4 Aims of the study

This exploratory study aims to investigate community expectations and attitudes towards teachers supporting student mental health in rural contexts using a mixed-method, cross-sectional design. Additionally, the study intends to commence developing a practical working definition of this role and consider what constitutes appropriate pre-service teacher mental health curriculum/training.

## 2 Method

### 2.1 Participants

Fifty-seven adults were recruited for the study using a convenience and snowball sampling approach. The researchers (1) emailed an invitation to participate in the study to contacts in their networks (i.e., convenience sample), and (2) asked potential participants to forward the email to their own contacts (i.e., snowball sampling). Given the lack of previous research in this area, there was no available data to inform statistical power calculations. As guidance on sample size for exploratory studies focuses on quantitative approaches (44), the authors aimed to recruit approximately 50 participants, while ensuring that a minimum of five participants (i.e., 10% of the total sample) were recruited from each participant category. When the sample reached over 50 responses and these were relatively evenly distributed between the groups, analysis was commenced. Due to difficulties using the same NVivo file inter-rater reliability was not calculated. Codes were generated and then compared between researchers.

Different community participant categories relevant to education settings were recruited: (1) parents and caregivers (*n* = 19, 33%), (2) community members^2^ (*n* = 13, 23%), (3) teachers (*n* = 17, 30%), and (4) mental health practitioners (*n* = 7, 12%). Participant ages ranged from 32 to 74 years (*M* = 52.81, *SD* = 9.57) of whom 43 (75%) identified as female and 13 (23%) identified as male. Participants were recruited from New South Wales, Australia (*n* = 51, 90%) and Victoria, Australia (*n* = 4, 7%), predominantly from regional and rural areas (*n* = 43, 75%). Fifty-four participants (95%) reported an English-speaking background. No participants reported an Aboriginal and/or Torres Strait Islander background. Fifty-eight percent (*n* = 33) of participants reported having postgraduate qualifications, 23% (*n* = 13) reported having undergraduate qualifications, 7% (*n* = 4) reported having vocational qualifications, and 4% (*n* = 2) reported having secondary qualifications.

### 2.2 Measures

Given this was an explorative pilot study, the employed measure was developed for the study and was non-standardised. Development of the measure was a posteriori and was based on the project team’s expertise and experience in psychology and education. Following initial development, the measure was pilot tested with a small (*n* = 5) focus group consisting of two school teachers and three community members. No changes were made to the instrument from this process.

The measure included demographic items, four quantitative Likert-scale questions (5-point), and eight qualitative items formatted as open-ended questions. As the Likert-scale questions measured different concepts it was not possible to calculate internal consistency for these items. An example of the Likert-scale questions used in the study is: “Should teachers be expected to do mental health work in schools to support students?” with responses ranging from 1 (definitely no) to 5 (definitely yes). An example of the qualitative questions used in the study is “How should teachers support students’ mental health in schools?”.

### 2.3 Data collection

Data were gathered via an anonymous online survey administered through SurveyMonkey (http://www.surveymonkey.com). Participants were provided with a link to the survey via the study’s participant information sheet. The study received institutional ethics approval from the Human Research Ethics Committee. Data was collected from 21 November 2023 to the 25 May 2024.

### 2.4 Data analysis

Quantitative analyses were conducted using Statistical Package for the Social Sciences 25 (SPSS). The authors considered the use of non-parametric group comparisons (specifically Fisher’s Exact Test). However, given the small sample size and non-random recruitment, we chose not to report inferential statistics as it is arguably problematic to generalise the results to the broader population. Consequently, quantitative analysis was limited to descriptive statistics (i.e., means, standard deviation, frequencies). The software package NVivo 14 was used to examine open-ended responses. Qualitative data analysis employed thematic analysis across, as well as within, each question. An inductive approach to thematic analysis was applied (45).

Data analysis commenced once the sample reached 50 participants. Sampling saturation sought even numbers for each category (sub-group) of participants. After familiarisation with participants’ responses, comments were collected into preliminary codes by two researchers and these were then compared and brought together to determine similarities and differences. The preliminary codes were refined by three researchers until the researchers were satisfied that the themes, codes, and raw data were closely aligned across the dataset (45). These were reviewed and agreed to by the other authors.

## 3 Results

The results present participant responses based on the order of questions in the survey. The first two questions involved examining quantitative descriptive data and the remaining questions required qualitative analysis focussed on providing a more nuanced understanding of participant perspectives.

### 3.1 Should teachers be expected to do mental health work in schools to support students?

Participants exhibited a varied response to this question with 57% of the sample responding probably or definitely yes, and 32% of the sample responding probably or definitely no (see **Table 1**).

**Table 1:** Participant attitudes to teachers being expected to do mental health work to support students.

Participants	Participant response				
	Definitely yes	Probably yes	Unsure	Probably no	Definitely no
Caregiver/Parents	5	5	2	2	1
Community members	3	4	1	3	2
Teacher	1	4	2	2	3
MH practitioners	3	2	0	2	0
Total: *n*(%)	12(25%)	15(32%)	5(11%)	9(19%)	6(13%)

*N* = 47. MH, mental health.

When participant category was considered, differences emerged. The majority of caregiver/parents (*n* = 10) and mental health practitioners (*n* = 5) reported that teachers should be expected to do mental health work in schools to support students. Community members were more evenly divided with a smaller majority (*n* = 7) answering definitely or probably yes. A slightly lower proportion (*n* = 5) of community members responded that teachers should be expected to do mental health work in schools to support students.

In contrast, teacher responses were more varied. A smaller proportion of teachers (*n* = 5) reported they should probably or definitely be expected to do mental health work in schools to support students. Five teachers indicated that they should probably or definitely not be expected to do mental health work in schools to support students. Two teachers reported being unsure about this issue which may reflect the varied nature of teacher responses to this question.

### 3.2 Are teachers adequately trained to support students’ mental health?

All stakeholders reported the view that teachers are not adequately trained to support students’ mental health, with a total of 95% of participants responding probably no (30%), or definitely no (65%). Three participants (5%) were unsure if teachers were adequately trained. **Table 2** provides details of participant responses.

**Table 2:** Participant attitudes about whether teachers are adequately trained to support students’ mental health.

Participants	Participant response				
	Definitely yes	Probably yes	Unsure	Probably no	Definitely no
Caregiver/Parents	0	0	1	7	7
Community members	0	0	2	3	8
Teacher	0	0	0	6	6
MH practitioners	0	0	0	3	4
Total: *n*(%)	0(0%)	0(0%)	3(6%)	19(40%)	25(53%)

*N* = 47. MH, mental health.

### 3.3 Responses to how teachers support students’ mental health in schools, how they should support students’ mental health, and the benefits and barriers of providing supports

All participants who completed the survey provided responses to open-ended questions. **Table 3** shows the number of responses to each question. The qualitative results are presented as themes emerging from participants’ responses.

**Table 3:** Number of participant responses to open-ended survey questions.

Questions	No. of responses
How do teachers support students’ mental health in schools?	101
How should teachers support students’ mental health in schools?	107
What benefits do teachers supporting students’ mental health in schools have for students and teachers?	67
What prevents teachers from supporting students’ mental health in schools?	130

#### 3.3.1 Referral to counsellors/qualified specialists

A major theme across all questions was that teachers support students’ mental health in schools through referrals to counsellors/qualified specialists. All groups (teachers, community members, mental health practitioners, and caregiver/parents) made comments acknowledging referrals to counsellors or mental health specialists as one way that teachers support students’ mental health in rural schools.

Accessing internal & external support services. (T#1^3^).

Knowing if/when to refer them on to other mental health supports is needed whether at school or outside of school. Providing students with information around access to relevant mental health support services as appropriate. (CGP#4).

Teachers are well placed to help identify and refer students with mental health issues to appropriate support. Early intervention. (MHP#7).

#### 3.3.2 Awareness of student mental health difficulties

Providing referral was often related to awareness of children having difficulties. All groups made comments endorsing teachers being aware and informed to support students’ mental health in rural schools:

There is mutual benefit to teachers and students (and the families of students) through an awareness and understanding of the mental health needs of students. (T#2).

By being aware something is different in the child that is in their school than how they typically present. (CGP#18).

An awareness of the human condition, including mental health education, would benefit the teacher’s management and support of their classroom and school environment. (CM#7).

Teachers can play an important role in supporting student mental health - awareness of potential mental health issues exhibited by students. (MHP#7).

#### 3.3.3 Understanding of mental health issues

Understanding of mental health issues was strongly emphasised by all groups and included an understanding of individual students, mental health issues related to children’s age, and a broader understanding of mental health:

Teachers should be aware of the common signs of mental health. (CGP#1).

For teachers, have a better understanding of how to help students who are struggling, know how to help the student work through their concerns. (CM#8).

Teachers will be prevented from supporting students if they do not understand the mental health issues of their students’ age group and how to deal with these issues. (CGP#9).

Through having a good understanding of what poor mental health/illness means, looks like, how it’s diagnosed and treated, and the impact it has on students/families lives (i.e. a good awareness of what it actually is). (T#9).

Teachers need an understanding of mental health to support students to remain in education. (MHP#4).

#### 3.3.4 Identifying mental health issues

Moving beyond understanding, there was an expectation that teachers be able to identify mental health issues which extended from “how to identify someone struggling” (CM#1) and “any potential issues” (CM#9) to behaviours and signs of poor mental health:

Teachers should know how to identify mental health issues and how to positively support the students. Whether the student is at a vulnerable stage in their lives or whether the student is stable they will always need their teacher to support them and guide them. (CGP#9).

Identifying anxiety, depression, withdrawing socially, extreme moods & actions that trigger red flags. (CGP#8).

#### 3.3.5 Promoting wellbeing

Community member, teacher and mental health practitioner comments linked mental health to wellbeing in supporting students in rural schools, “where teachers practise and explicitly model” wellbeing (T#3) and engage in “psycho-education” (MHP#3):

Ensuring the general well-being for all students. (CGP15).

Take regular observations of well-being. (T16).

Specific strategies to build into the classroom environment. Some focus on their own well-being and ways to care for self while also caring for students in this space. (MHP6).

#### 3.3.6 Teacher-student relationships

Teachers, community members, and mental health practitioners made comments on the importance of teacher-student relationships in supporting students’ mental health:

Listening, showing care, providing safe places. (CM#1).

The more the teacher is able to relate to the students and support them, then the more positive the relationship will be. The student will feel valued and hence be more open to learning. (CGP9).

Positive relationship building, develops trust and respect for staff and students. Creates a welcoming, inclusive and safe space for learning. Teachers feel an important connection with students. (T16).

Class teachers can act as a safe adult and build rapport with students. (MHP2).

#### 3.3.7 Communication and listening with empathy

Teachers, mental health practitioners, caregiver/parents, and one community member recognised communication and listening with empathy as a way that teachers should support students’ mental health in rural schools.

Ensure open communication to ensure children are comfortable in having discussions with teachers about sensitive topics. (CGP#11).

Teachers should demonstrate empathy, understanding, compassion, acceptance, and they should make provision for students with a wide range of health and social issues in the ways in which they teach and interact with students and their families. (T#2).

#### 3.3.8 Considering student needs

Some participants recognised the importance of considering student needs and not just errant behaviour:

All teachers should be trained in different mental health conditions and how to recognise it and adjust learning plans accordingly. I don’t believe it’s all on the shoulders of teachers but even something as simple of being aware and noticing something and being proactive to reach out to the parent to work with them to give that student the best chance at learning. Not just writing them off as the naughty kid (CGP#2).

Having mental health support strategies to employ when needed in class/school to help students before they can access professional mental health support. (T#9).

#### 3.3.9 Student safety

Several comments were made that teachers support students’ mental health in rural schools by providing a safe environment, being a safe adult, and building rapport as well as addressing learning needs:

One other thing that should go without saying (but sadly often doesn’t)! is that teachers should support students’ mental health by not adding to their burden - by creating a safe, calm, predictable learning environment and being a safe, calm, trusted person who does not overload their students with unnecessary pressures (T#3).

Teacher roles in mental health can only be generalised to creating a safe learning space and positive atmosphere, with the ability to recognise potential and existing issues and knowledge of what support services they can link children to - and the ability to easily link children to appropriate services. (T#10).

Students do not learn when they do not have a safe, calm, predictable learning environment - especially those students with mental health issues. As teachers, our core business is teaching and learning, but we must create the optimal environment for learning to happen - of course these benefits both teachers and students. (T#3).

#### 3.3.10 Limited expectations

Some respondents noted clear limitations regarding teachers’ mental health knowledge and comments recognised potential limits on expectations:

Teachers have a duty of care to support student learning and definitely should address needs in relation to that. However, they are not mental health professionals. (T#12).

Teachers are often asked to provide a level of support for student mental health issues that is beyond their training to do. Teachers may be asked to engage in crisis management, deal with students with various mental health issues, and implement mental health interventions. (MHP#7).

Comments recognised a “teacher’s role is to teach, not to be a counsellor but to provide referrals to counsellors” (CM#12) and appeals for “more counsellors at schools and let teachers teach” (T#13):

Teachers build a positive rapport with students and then teach - not be a counsellor, or health worker. (T#1).

The teacher’s responsibility was distinguished from other professionals by some comments:

Teachers should only do general support by creating a positive and safe learning space. Qualified psychologists should be attached to each school in sufficient numbers as school counsellors. (T#10).

Teachers are at schools to teach all students equally that want to learn. Mental health issues are not the teachers’ responsibility to manage. (CM#10).

#### 3.3.11 Perceived barriers

Participants commented that inadequate training, time, funding, workload pressure, and a lack of role clarity in the mental health space prevented teachers from supporting students’ mental health in rural schools:

Already stretched and under resourced. (CGP#15).

Not enough training to identify, and support students with mental health. Not enough training for teachers to support their own mental health. (CGP#1).

Time. Teachers are overloaded with their teaching and have no time for students’ mental health and wellbeing”. (CGP#16).

Large numbers in classrooms and behaviour management issues in some classes. (CGP#18).

Sometimes the mental illness is not disclosed. (T#14).

One comment made connections between teachers’ knowledge, time, and workload:

Limited understanding, limited time, limited resources. These three things are quite intrinsically linked. Limited understanding often leads to an inflexibility - a refusal to make any allowances/treating all students the same/focussing solely on the learning and ticking off outcomes (‘Getting through the curriculum’), rather than the learners. Limited time sometimes means teachers feel unable to support learners in the ways they know they should. Limited resources contribute to limited understanding (training) and time.” (T#3).

Teachers also recognised a lack of support from allied health professionals and that this could impact their confidence:

Lack of support from trained mental health professionals such as psychologists, behaviour support specialists and early intervention services such as Occupational Therapy and Speech Therapy. (T#8).

Lack of confidence due to lack of professional resourcing and support. (CM#12).

Finally, lack of clarity regarding the teachers’ role and responsibilities in the mental health space was viewed as a significant barrier to supporting students:

Not interested (i.e., perception it is not their job). (MHP#7).

If teachers don’t see this as part of their role or responsibility at school, they may not feel comfortable to support students in this way. (CGP#4).

## 4 Discussion

Community attitudes and expectations towards teachers’ roles in supporting student mental health have been underexplored despite schools being positioned as a setting for promoting mental health and wellbeing. Participants in this study did not consistently support the idea that teachers should be expected to do mental health work in rural schools to support students. While a larger proportion of participants (57%) indicated that teachers probably or definitely should support student mental health, 32% indicated that teachers probably or definitely should not. Differences in attitudes across participant categories were observed, with fewer teachers and more parents, caregivers, and mental health practitioners reporting that teachers should support student mental health. Community members were more evenly divided. These results provide some support for the claim that the community expects school teachers to perform frontline mental health provider roles (4). However, this was not a consistent view among participants.

Conversely, participants clearly reported the view that teachers were not adequately trained to perform mental health work with students. It is noteworthy that no participants reported that teachers were adequately trained to support student mental health. This is perhaps unsurprising given that pre-service teacher education programs may not explicitly address mental health training (37) and that in-service teachers report low confidence in supporting student mental health (39).

The study’s qualitative findings offer useful directions for beginning to develop a practical working definition of the role teachers might take in supporting student mental health. Many participants supported the idea that teaching is a distinct role from providing mental health support for students. Teachers were not considered to be mental health specialists or adequately trained to support mental health. Despite this, the current results provide some support for the view that the community believes teachers should provide mental health support for rural students. This may result from (1) underfunding of mental health and psychiatric care (3), (2) inadequate numbers of mental health professionals especially in rural settings (29), in (3) combination with the convenience that schools provide almost universal access to youth (31). While *community expectations* may be apparent, the question of whether and how teachers should perform mental health roles remains.

One possible direction suggested by the results is that teachers should provide support and address the needs of students as learners, which underpins wellbeing in school environments. The instructional role of teachers provides opportunities to support student mental health without assuming the role of a frontline mental health provider (36). Specific strategies that teachers routinely implement that support mental health can include: (1) developing positive learning environments catering to individual student needs and strengths, (2) providing opportunities to build positive student identity and self-esteem by providing genuine opportunities for students to succeed in the classroom, (3) promoting positive relationships with their students and foster healthy peer interactions, and (4) referring students to mental health services for specialist support (41). While the extent and type of mental health knowledge needed by teachers has not been defined and remains unclear, the results indicate that greater teacher knowledge is required in the areas of mental health awareness and referral. However, this should be balanced with consideration of how much training is appropriate from a professional perspective. As respondents noted, “teachers might be a good interim measure to support student mental health” (CM#11), but “they are not mental health professionals” (T#12).

These results offer a range of community perspectives towards teachers supporting student mental health in Australian rural contexts, however the following limitations of the study should be noted. The study’s design was cross-sectional and is limited by a single data collection timepoint. The sample size was small and participants exhibited a highly educated background. These sample characteristics may bias the study’s results. The underrepresentation of culturally and linguistically diverse groups and the absence of Aboriginal and Torres Strait Islander participants should also be noted in this regard. While arguably suitable for a pilot study, the use of a non-standardised, study-specific instrument is a major limitation. Consequently, generalisations from the study should be made cautiously and with reference to the research design, sample, and methodology.

### 4.1 Future directions

Future research arising from this study will (1) examine community expectations of teachers and mental health roles using a longitudinal research design with greater participant numbers across Australian and international settings, (2) examine rural and urban contexts, and (3) develop a self-efficacy scale examining perceived capacity for teachers (and others) to conduct mental health work. Additionally, (4) the research will review Australian tertiary institutions’ mental health curriculum/training for pre-service teachers, and (5) develop and pilot an empirically-based mental health training program for pre-service teachers. The scope of this work will significantly address the limitations of the current study.

## 5 Conclusion

The results of this pilot study suggest the *community expectation* of teachers providing mental health services in rural schools is not consistently supported. However, the results clearly indicated that different stakeholders believed teachers were not adequately trained to perform mental health work with students. While a similar pattern of results from future work with larger sample sizes is expected, it is premature to make an informed conclusion given the exploratory nature of these results. The study provides some support for the view that teachers have a role in supporting students’ mental health in schools. However, this is not a teacher’s key role and teachers are not adequately trained to do this. Further clarification of the expectations of teachers, their role, and their responsibilities in supporting students’ mental health in schools is needed.

## Data Availability

The raw data supporting the conclusions of this article will be made available by the authors, without undue reservation.

## Footnotes

1 The Black Dog Institute is an Australian not for profit mental health research institute.

2 Community members were defined as any member of the community who did not identify with the other participant categories.

3 T= Teachers; CGP = Caregiver/parent; MHP = Mental health practitioner; CM = Community member.
